# Design Methodology of a Dual-Halbach Array Linear Actuator with Thermal-Electromagnetic Coupling

**DOI:** 10.3390/s16030360

**Published:** 2016-03-11

**Authors:** Paulo Roberto Eckert, Aly Ferreira Flores Filho, Eduardo Perondi, Jeferson Ferri, Evandro Goltz

**Affiliations:** 1 Post-Graduate Program in Electrical Engineering, Federal University of Rio Grande do Sul, Av. Osvaldo Aranha 103, Porto Alegre, RS 90035-190, Brazil; aly.flores@ufrgs.br; 2 Department of Mechanical Engineering, Federal University of Rio Grande do Sul, Rua Sarmento Leite 425, Porto Alegre, RS 90050-170, Brazil; eduardo.perondi@ufrgs.br (E.P.); jeferson.ferri@ufrgs.br (J.F.); 3 Technology Centre, Federal University of Santa Maria, Av. Roraima 1000, Santa Maria, RS 97105-900, Brazil; evandro@inf.ufsm.br

**Keywords:** design methodology, dual quasi-Halbach actuator, linear actuators design, moving-coil actuator, parametric analysis, thermal-electromagnetic coupling, tubular actuators

## Abstract

This paper proposes a design methodology for linear actuators, considering thermal and electromagnetic coupling with geometrical and temperature constraints, that maximizes force density and minimizes force ripple. The method allows defining an actuator for given specifications in a step-by-step way so that requirements are met and the temperature within the device is maintained under or equal to its maximum allowed for continuous operation. According to the proposed method, the electromagnetic and thermal models are built with quasi-static parametric finite element models. The methodology was successfully applied to the design of a linear cylindrical actuator with a dual quasi-Halbach array of permanent magnets and a moving-coil. The actuator can produce an axial force of 120 N and a stroke of 80 mm. The paper also presents a comparative analysis between results obtained considering only an electromagnetic model and the thermal-electromagnetic coupled model. This comparison shows that the final designs for both cases differ significantly, especially regarding its active volume and its electrical and magnetic loading. Although in this paper the methodology was employed to design a specific actuator, its structure can be used to design a wide range of linear devices if the parametric models are adjusted for each particular actuator.

## 1. Introduction

The advantages of linear actuators over rotation-to-translation conversion systems when linear motion is needed are well documented in the literature [1]. Applications of linear actuators are mainly in industry and transportation due to its force density, efficiency, levels of acceleration, and precision. An application that has also drawn particular interest to linear actuators is mechanical vibration control [2,3]. For such an application, linear actuators that have interesting characteristics are the cylindrical actuators equipped with quasi-Halbach arrays of permanent magnets (PMs) [4,5]. The advantages of quasi-Halbach arrays are well known, and include applicability for a wide range of electromechanical devices [6]. Even so, there are still recent developments and designs for specific applications under research, such as, for example, for limited angle torque actuators [7] and for assemblies in which the flux density can be altered by a mechanical operation [8].

**Figure 1:**
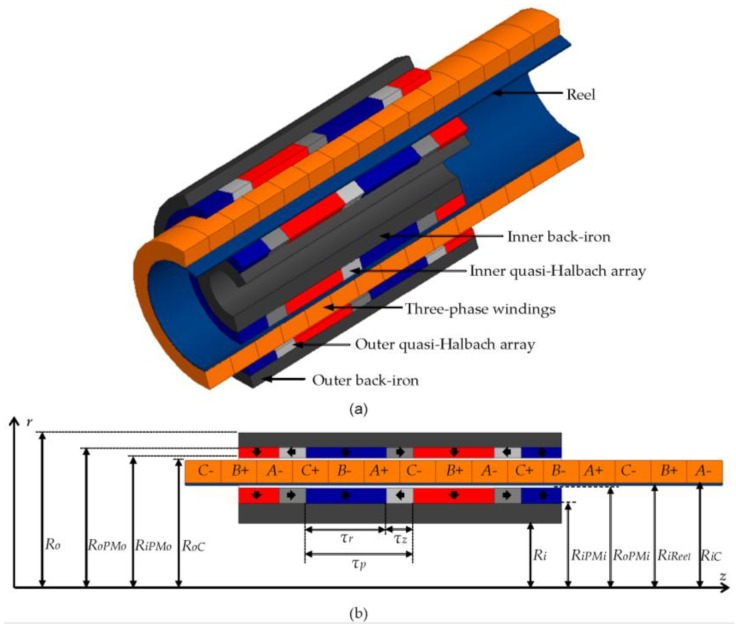
Linear tubular moving-coil actuator with a four pole dual quasi-Halbach array: (**a**) 3D isometric section view indicating structural elements; and (**b**) 2D half-section indicating design variables.

In this context, this paper proposes a methodology applied to design a special cylindrical actuator with dual quasi-Halbach arrays and a moving-coil coreless armature that was first proposed by Yan *et al.* [9]. Figure 1a shows a 3D view of the constitutive elements of the actuator. It consists of a device with a stator formed by two layers of PMs mounted on the surface of the inner and outer back-irons, in order to produce two concentric quasi-Halbach arrays, in accordance with the magnetization pattern shown by the arrows inside the PMs in Figure 1b. Thus, a multipolar distribution of the magnetic field in the axial direction in the air-gap volume occupied by the moving-coil is produced. The three-phase *ABC* windings are supported mechanically by a reel, forming a coil concentrically mounted in relation to the quasi-Halbach arrays and having free relative movement in the axial direction. The three-phase currents in the windings generate a multipolar magnetic field distribution in the axial direction, analogous to the one produced by the PMs. When magnetic fields produced by PMs and currents in the three-phase windings are in quadrature, the electromagnetic force produced in the axial direction is maximized.

With regard to the PMs of the quasi-Halbach arrays, the ones with axial magnetization can be easily manufactured; however, the ones with radial magnetization, when using NdFeB sintered material, *i.e.*, with high *B*-*H* product, should be segmented and magnetized in a parallel direction to avoid a more complicated and expensive magnetizing fixture. In [10], it was shown that such a method slightly reduces the performance if the ring is segmented with an appropriate number of elements.

Some features of the actuator presented in Figure 1 are attractive when compared to other permanent magnet tubular actuators, such as, for example: the moving-coil reduces the moving mass of the actuator, increasing acceleration and speed; there is no cogging force, considering that there are no slots, which significantly contributes to reducing the force ripple; core losses are zero because it is a coreless device, leading to higher efficiency; the quasi-Halbach array on both sides of the windings increases magnetic flux density and allows for a distribution with low harmonic distortion in the magnetic gap, which increases force density and reduces ripple of force, respectively. The high levels of flux density can be explained by the fact that the quasi-Halbach array reduces local leakage flux, *i.e.*, from PMs to back-iron, while the double array helps to decrease interpolar leakage flux. In [11] the authors show that if the same volume of PMs is employed by this topology, in comparison to a topology with outer or inner PMs only, the dual PMs array provides a better force density.

Design parameters of the topology presented in Figure 1 were discussed in [12], where the authors concluded, based on an analytical model, that the topology has better force capability and lower force ripple with three-phase windings than ones with one-phase or with two-phase windings. Some geometrical analyses were performed based on an analytical electromagnetic model.

In [13], a thermal-electromagnetic coupled design methodology is presented for the project of a short-duty slotted non-Halbach array with axially magnetized PMs linear actuator. The duty cycle of the actuator required a transient thermal analysis because high levels of transitory current density were employed.

Designs of specific actuators that consider thermal influence are addressed in the literature, for example, in [14,15]. In [14], a coupled thermal-electromagnetic analysis of slotless tubular permanent magnet machines was presented. Bianchi *et al.* [15], on the other hand, presented an overall comparison on linear actuators such as interior and surface-mounted PMs, and slotted and slotless motors. These two references apply a thermal constraint as maximum temperature of the actuator; thermal influence is inserted as a constant overall heat transfer coefficient.

In [16] a multiphysics approach to model a tubular permanent magnetic slotted actuator with radially magnetized non-Halbach and interior surface mounted PMs based on finite element models is addressed. Encica *et al.* [17] described an optimization methodology employed in the design of an actuator with a similar topology as in [16] using thermal and electromagnetic lumped circuit models.

The development of a design methodology for electromagnetic devices using modern computational tools is an extensive task that involves a coupling between the electromagnetic and thermal domains. However, one can expect a high degree of conformity between models and the results obtained experimentally.

In this paper, a design methodology that encompasses modeling and analysis where specifications such as force and stroke must be met considering thermal-electromagnetic coupling for continuous operation is addressed. Specifications are met with maximization of force density and minimization of force ripple subjected to geometrical and thermal constraints. Thermal and electromagnetic 2D axisymmetric models are built and simulated with finite element software packages. A post-processing analysis is carried out based on results of parametric electromagnetic simulation.

The proposed methodology can be employed to design a wide range of linear devices, such as step motors, synchronous machines, DC machines, and reluctance machines. In order to employ it in different devices, the electromagnetic parametric model and thermal model must be particular to each case. The main requirement to apply this methodology is that the linear actuator should be a multipole device with long armature, although it could be adapted to short armature if the steps that compute the number of poles and axial length are adjusted. However, the proposed methodology cannot be directly employed to design linear single-phase oscillatory actuators, linear plunger solenoids, and single-phase solenoids, for instance, because those are examples of non-multipolar devices.

## 2. Design Methodology

**Figure 2:**
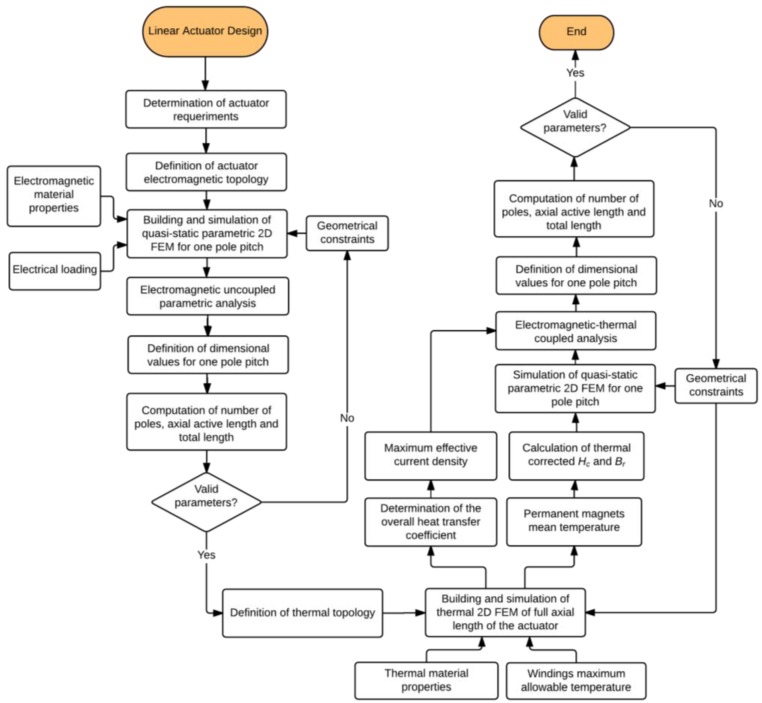
Flowchart of the proposed design methodology of linear cylindrical actuators with electromagnetic-thermal coupling.

The first step in developing the proposed design methodology is the rationalization of an engineering problem considering a specific application that can be obtained by a flowchart, as shown in Figure 2. Identifying the technical requirements of axial force and the stroke of an actuator associated with dimensional constraints are the main input elements for design. The main goal is to obtain the geometric relationships of the device, in order to maximize force density and minimize force ripple.

The proposed design methodology is mainly based on obtaining an absolute value for the force density of one pole pitch of the device, finding its best design with the aid of parametric analysis, and, afterwards, determining the actuator geometrical relationships and dimensions that are able to meet the design specifications.

An initial approach is proposed in which a design is obtained from a pure electromagnetic model, in this paper referred to as “uncoupled model”. This model provides initial geometric dimensions to allow for a simulation of the thermal model. A thermal constraint, in the form of a maximum allowable temperature at the armature, is applied, which is the basis for adjusting the effective current density accordingly, and the operation characteristics of PMs for the so called “coupled model”.

A similar approach could be conducted with a multiphysics thermal-electromagnetic coupled simulation by means of finite element models for both domains, which is already available in some commercial packages. However, such an analysis would be an extensive and time-consuming task and it does not provide a routine to design a device.

## 3. Design of the Cylindrical Linear Actuator with Dual-Halbach Array

In this section, each of the design steps presented at the flowchart of Figure 2 is discussed in detail. The steps are followed in order to design a specific cylindrical linear actuator, which is used as a case study.

### 3.1. Determination of Actuator Requirements

This is the first step in the design process, *i.e.*, identifying which are the output parameters that the linear actuator has to meet. In the design of electrical rotating machines, the first specification is generally the rated torque, which allows us to determine the active volume once electrical and magnetic loading are specified [18]. For conventional rotating machines, loading characteristics are available in the references, such as [18], based on extensive empirical data available or resultant from heat transfer analysis. However, in linear machines with unconventional topologies and complex heat transfer systems, scarce information is available, especially about the compromise between electrical loading and heat exchange ability depending on the temperature constraints of the materials.

In the case of linear machines, similar to rotating ones, the rated effective axial force that the actuator has to produce in continuous operation must be specified. It is important to highlight that short-stroke linear actuators hardly operate with constant speed, different from rotating machines; therefore, the axial force they produce may also vary. In this sense, it is important to point out that effective force must be set as rated, once it is directly proportional to the effective current density, to which the main source of losses is quadratically proportional, *i.e.*, Joule losses, imposing a performance limited by temperature.

Another essential output parameter that must be specified is the stroke of the actuator, which is required to determine the axial length of armature *L_zW_* and the total length of the actuator *L_zT_*.

In this paper, the techniques for specification of force and stroke will not be discussed; instead, an actuator will be designed for a general application that requires a rated effective axial force *F_r_* of 120 N and stroke *S* of 80 mm.

### 3.2. Definition of Actuator Electromagnetic Topology

Although the general idea of the proposed methodology is applicable for the design of a wide range of linear actuator, there are particularities applied to the parametrization, as presented in Section 3.3, and to the thermal and electromagnetic models for one pole pitch, *τ_p_*, which are exclusive to each linear actuator topology. For this reason, the definition of the actuator topology must be a step in the design process. The selection of the topology can depend on the application, on actuator performance or cost, *etc*. In this paper, a cylindrical actuator with a three-phase moving coil and two arrays of quasi-Halbach PMs that has some interesting features is addressed.

### 3.3. Building and Simulation of Quasi-Static Parametric 2D FEM for One Pole Pitch

The parameterization of the model is essential, once it enables variation of the dimensional parameters that define the topology of the electromagnetic actuator. By means of this geometric model and the results of numerical parametric simulation, it is possible to analyze the behavior of the various figures of merit for the device performance in terms of interdependency and sensitivity of variables.

The first step in building this model is the identification of the geometric variables that define the topology, as shown by Figure 1. The active volume of one pole pitch is delimited by *R_iPMi_*, *R_oPMo_* (the inner and the outer radii, respectively), and *τ_p_*. Some variables have dimensional constraints related to manufacturing issues of the parts or to limitation of space in a specific application. Ring-shaped PMs have a limitation for both *R_iPMi_* and *R_oPMo_*, due to the need for radial magnetization and the dimensional limitations of the magnetizer. Even though techniques of segmentation of the rings and parallel magnetization are employed [10], in this particular case there is a need for a gas mass flow channel for cooling the device, which also requires *R_iPMi_* > *0*. The thermal heat transfer mechanism is discussed in Section 3.8.

The axial length of the PMs with radial magnetization, *τ_r_*, may be normalized according to the pole pitch *τ_p_*, *i.e.*, *τ_r_*/*τ_p_;* therefore, the axial length of the PMs with axial magnetization, *τ_z_*, is

(1) $$\tau_{z}=\left(1-\frac{\tau_{r}}{\tau_{p}}\right)\tau_{p}$$

In a cylindrical device of finite length, it is possible to observe a relationship between the axial length and the diameter. This relation can be named as form factor, and, in this case, it is given by the ratio of the pole pitch to the external radius of outer PMs, according to

(2) $$n_{form}=\frac{\tau_{p}}{R_{oPMo}}$$

Due to the aspects previously mentioned, it is interesting to design the actuator using a hollow shaft. Therefore, the form factor must be set to allow devices with annular cross sections. The cylindrical case results when *R_iPMi_* = *0*, which excludes the possibility of radial magnetization and cooling air flow. To maintain consistency in the definition of the form factor, volumetric comparison is required between the cylindrical and ring cases, which results in an equivalent radius and provides a proper definition for the form factor given by

(3) $$n_{form}=\frac{\tau_{p}}{\sqrt{\left({R_{oPMo}}^{2}-{R_{iPMi}}^{2}\right)}}$$

The result of the expression is dimensionless and provides a comparison of devices with different scales. The form factor is suitable for the use as a parametric variable because it condenses all the dimensional variables that define the active volume for one pole pitch of the device.

A parametric variable that is defined as the ratio between the radial length of the coil *L_rCoil_* and the radial active space, *i.e.*,

(4) $$N_{CPMs}=\frac{L_{rCoil}}{\left(R_{oPMo}-R_{iPMi}\right)}$$

Another parametric variable is defined as the ratio between the radial length of the inner PMs and the total radial length of PMs:

(5) $$N_{PMi}=\frac{L_{rPMi}}{\left(L_{rPMi}+L_{rPMo}\right)}$$

where *L_rPMi_* = (*R_oPMi_* − *R_iPMi_*), and *L_rPMo_* is the radial length of the outer PMs, *i.e.*, (*R_oPMo_* − *R_iPMo_*).

A parametric variable that affects only the radial length of the back-irons is given by

(6) $$n_{BI}=\frac{{R_{iPMi}}^{2}-{R_{i}}^{2}}{R_{iPMi}\tau_{r}}=\frac{{R_{o}}^{2}-{R_{oPMo}}^{2}}{R_{oPMo}\tau_{r}}$$

This variable relates the cross section area of the back-irons with half of the cylindrical surface area of PMs with radial magnetization in contact with the back-irons. If *n_BI_* = 1, the areas mentioned are equal to each other.

The *n_BI_* factor can be initially estimated by magnetic properties of materials. For devices with coreless armatures and quasi-Halbach arrays, a good rule is

(7) $$\frac{B_{r}}{2B_{sat}}\leq n_{BI}\leq \frac{B_{r}}{B_{sat}}$$

where *B_r_* is the remanent maximum flux density of the PMs and *B_sat_* is the saturation flux density of back-iron soft ferromagnetic material. Because the topology uses quasi-Halbach arrays, the influence of back-irons in the device performance is less significant in comparison to actuators with non-Halbach arrays. Simulation results of flux density in the back-iron regions must be analyzed and the radial length adjusted in order to avoid saturation.

Considering a position-dependent synchronous electric drive, e.g., with pulse width modulation, the sinusoidal currents of excitation in the sections of the windings are defined according to

(8) IA=2JrmsAcondFf sin(θe)IB=2JrmsAcondFf sin(θe+2π3)IC=2JrmsAcondFf sin(θe−2π3)

where *J_rms_* is the effective current density through the copper conduction area, *A_cond_* = *τ_p_L_rCoil_*/*3* is the geometric conduction area of one coil in the model, *F_f_* is the fill factor of the windings, and *θ_e_* is the position-dependent electrical angle in radians. By means of these equations, the current density applied to a FEM produces the same magnetomotive force that would be observed in the experimental case.

Considering a study in the quasi-static domain, it is possible to study the behavior of the electromagnetic force depending on the relative position between the stator and the translator, *p_z_*, which can be defined in terms of the electrical angle according to

(9) $$p_{z}\left(\theta_{e}\right)=\frac{\tau_{p}\theta_{e}}{\pi }$$

By means of Equations (8) and (9), the initial relative position *p_z_*(0) corresponds to the electrical angle at which the current in phase *A* is zero, but the currents in phases *B* and *C* are identical, so the coil must be positioned with alignment of the phases *B* and *C* with the pole faces of the PMs with radial magnetization and axial length *τ_r_*. Therefore, the generated fields are in quadrature, producing the maximum force in relation to the applied current.

Using equations presented in this subsection, it is possible to build parametric models of the geometry in 2D or 3D spaces. The geometry is axisymmetric, so 2D models satisfactorily represent the behavior of the spatial distribution of the fields, even if the technique of segmenting the rings into a minimum of eight sections with parallel magnetization is applied [10]. However, if the number of segments is lower than eight, and the asymmetry in the circumferential direction becomes relevant, the 2D model must be replaced by a 3D model. Thus, the model would be more complex and simulation would become time consuming, but the main structure of the proposed methodology would still be applicable.

In order to create and simulate the 2D FEM for one pole pitch, the commercial package Ansys Maxwell^®^ V16.2.0 was employed. The boundary conditions at the axial boundaries of the model were set as symmetric Master/Slave, *i.e.*, *B_slave_* = −*B_master_*. Thus, the spatial distribution of the field in these regions behaves as if they had infinite neighboring poles. This approach neglects end effects, however; at this stage, the number of poles of the device to be designed is not known, so adverse effects should be avoided.

The number of poles needed to meet the force requirement in continuous operation is computed in Section 3.6 and Section 3.17. The magnetic end poles are set as radial magnetized PMs with half the axial length, *i.e.*, the end poles present radial length *τ_r_*/2 in order to minimize back-iron flux.

**Figure 3:**
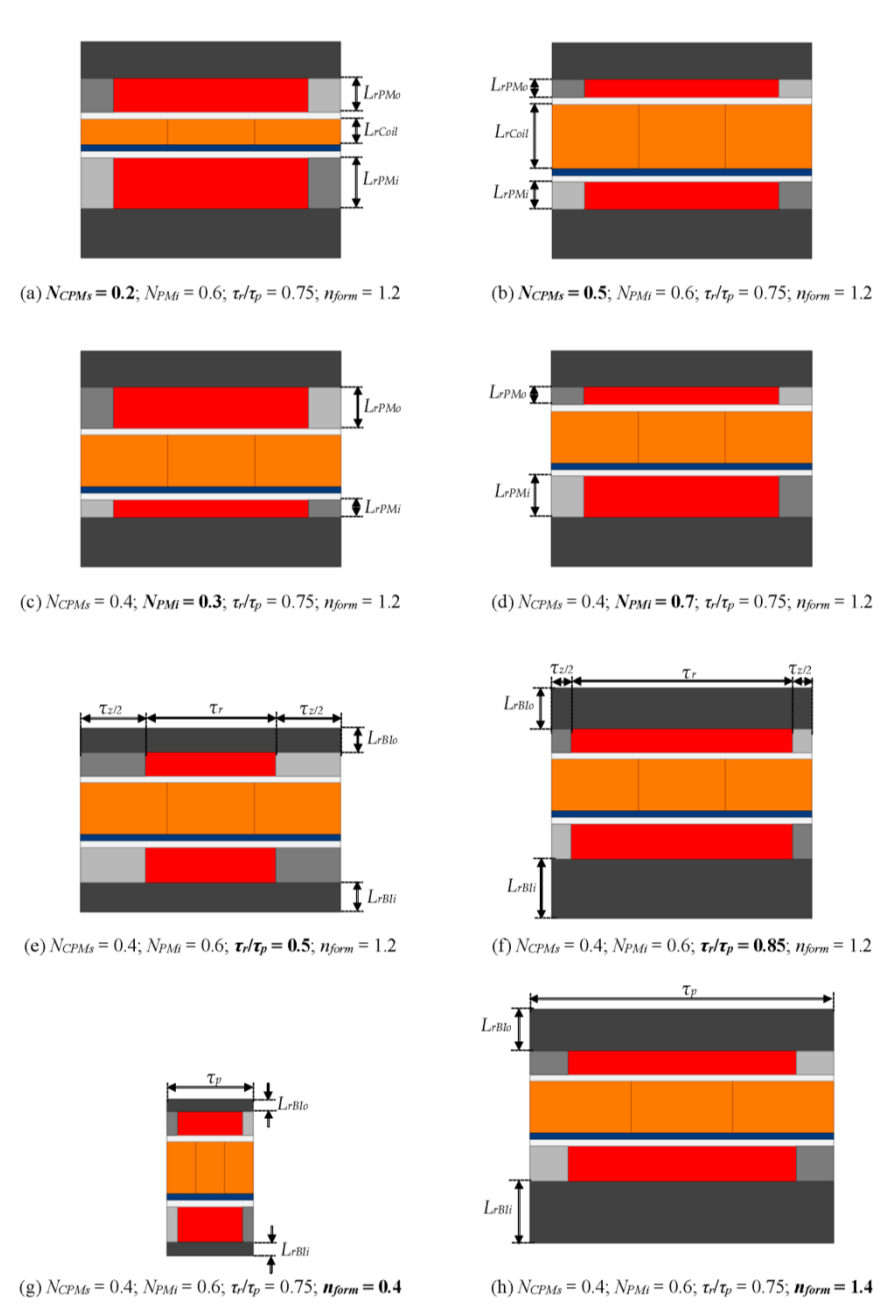
2D parametric models of one pole pitch presenting limits of parametric variation according to Table 1: Variations (**a**) *N_CMPs_* = 0.2; (**b**) *N_CPMs_* = 0.5; (**c**) *N_PMi_* = 0.3; (**d**) *N_PMi_* = 0.7; (**e**) *τ_r_*/*τ_p_* = 0.5; (**f**) *τ_r_*/*τ_p_* = 0.85; (**g**) *n_form_* = 0.4; and (**h**) *n_form_* = 1.4. While one parametric variable was varied the others were kept constant with *N_CPMs_* = 0.4, *N_PMi_* = 0.6, *τ_r_*/*τ_p_* = 0.75, and *n_form_* = 1.2. The variable *n_BI_* was also kept constant and equals 0.4 in all cases.

Table 1 presents the design variables, and Figure 3 shows the influence of the four parametric variables with variation between its lower and upper limits, according to the evaluated values.

In Figure 3, the geometric dimensions affected by the parametric variables highlighted in bold below its respective figure are indicated. The parametric variable *N_CPMs_* affects the radial length of the coils *L_rCoil_* and the radial length of the inner and outer arrays of PMs, *L_rPMi_* and *L_rPMo_*, respectively. The radial lengths of the PMs are also altered by *N_PMi_*. The parametric variable given by the ratio *τ_r_*/*τ_p_* has effect over the radial length of the inner back-iron *L_rPMi_*, the radial length of the outer back-iron *L_rPMo_*, the axial length of the radially magnetized PMs *τ_r_*, and of the axially magnetized PMs *τ_a_*. The radial lengths of the back-irons are also a function of *n_form_*. In addition, *τ_p_* is also affected by *n_form_*.

#### 3.3.1. Electromagnetic Material Properties

The electromagnetic material properties should correspond to the properties of materials employed in the construction of the actuator, if such data are available. In this case study, the properties were defined as default by Ansys Maxwell^®^ library, with values at a temperature of 25 °C, once the main scope of this paper is about the design methodology itself, and not the comparison with experimental results at this stage.

The main material properties are as follows: PMs made of sintered NdFeB with nominal maximum energy product of 278 kJ/m^3^, *B_r_*(*T_0_*) = 1.23 T, *H_c_*(*T_0_*) = −890 kA/m, where *T_0_* is the temperature of 25 °C, and *µ_r_* = 1.099; back-iron pieces were set with nonlinear *B-H* curve of steel 1010 with *B_sat_* = 2.0 T; coil made of annealed copper with bulk conductivity $\sigma_{cooper}=\;5.8\;\times \;10^{7}$ S/m and *µ_r_* = 0.999991; and the reel was set to use a high-temperature polymeric material, named Teflon^®^, with *µ_r_* = 1 and zero conductivity.

#### 3.3.2. Electrical Loading

The initial electrical loading should be an estimation of what effective current density could be applicable in continuous operation mode. For conventional rotating machines, this is available in the references, e.g., [18], but for linear actuators with more complex heat transfer systems this is hardly known, if no appropriate study were carried out. In this case, it was assumed that the effective current density is similar to the one used in electrical machines with natural convection, *i.e.*, *J_rms_* = 3 A/mm^2^.

#### 3.3.3. Geometrical Constraints

The geometrical constraints applied to this specific case study are summarized in Table 2.

Ring-shaped PMs have limitations applied to the inner radius of the internal array *R_iPMi_* and to the outer radius of external array *R_oPMo_*. By assigning a constant value to these variables, the active region to be analyzed is limited radially.

The limitation applied to the *R_oPMo_* can be justified due to restrictions of physical space where the actuator must be installed or due to magnetizer restriction. In this work, it was set considering the limitation imposed by the magnetizer fixture available, *i.e.*, model X-Series from Magnet Physik^®^, which presents a maximum outer radius of approximately 38 mm.

The limitation imposed on *R_iPMi_* is justified by the need for radial magnetization and to enable air flow for cooling of the actuator. The radial magnetization on ring-shaped PMs would be physically impractical on a cylinder if *R_iPMi_* is zero; therefore, a limitation imposed by the magnetizer fixture available, if ideal radial magnetization is desired, may apply. On the other hand, to enable air flow passing through a hollow shaft in the inner back-iron, discussed in Section 3.8, with a transversal area with the same order of magnitude of the transversal area of the mechanical air-gaps, requires *R_iPMi_* to be estimated accordingly. In this case study, it is considered that radially magnetized PMs are active with segmentation and parallel magnetization [10], so that no restriction by magnetizer fixture is directly applicable. Thus, considering the *R_oPMo_* dimension, and the needed air flow, *R_iPMi_* was initially set as 18 mm; however, if necessary, *R_iPMi_* could be reconsidered during the project.

It should be noted that even if segmentation and parallel magnetization are also applied to the PMs of the outer array, restriction of *R_oPMo_* imposed by the magnetizer is still applicable in order to manufacture the axially magnetized PMs in a ring shape without segmentation.

The mechanical gaps depend on many factors, such as bearing backlash, manufacturing tolerances, thermal expansion of materials, *etc*. In this paper, inner and outer gaps were set as constant with a value that is typically found in the literature and in datasheets of manufactures and suppliers of linear actuators; however, such value might be minimized, if an in-depth study about the factors that affects mechanical gaps were performed.

The radial length of the reel should be as short as possible, once its presence increases the magnetic gap. In this topology, the reel is necessary to provide more stiffness to the moving coils, and its radial length was defined based on simulation of mechanical stress.

The constraints applied to the radial lengths of the PMs are discussed in detail in Section 3.7.

The back-iron factor was set in order to satisfy the condition given by Equation (7); thus, all simulations considered *n_BI_* = 0.4. If desired, after defining all other variables, *n_BI_* could be optimized in order to possibly reduce some of ferromagnetic material of the inner and outer back-iron pieces.

Observing the flowchart presented in Figure 2, it is possible to see that if some conditions, addressed in Section 3.7, are not met, new geometrical constraints must be applied. These new geometrical constraints may apply especially to *R_oPMo_* or to *R_iPMi_*, depending on how flexible each one of those is.

### 3.4. Electromagnetic Uncoupled Parametric Analysis

**Figure 4:**
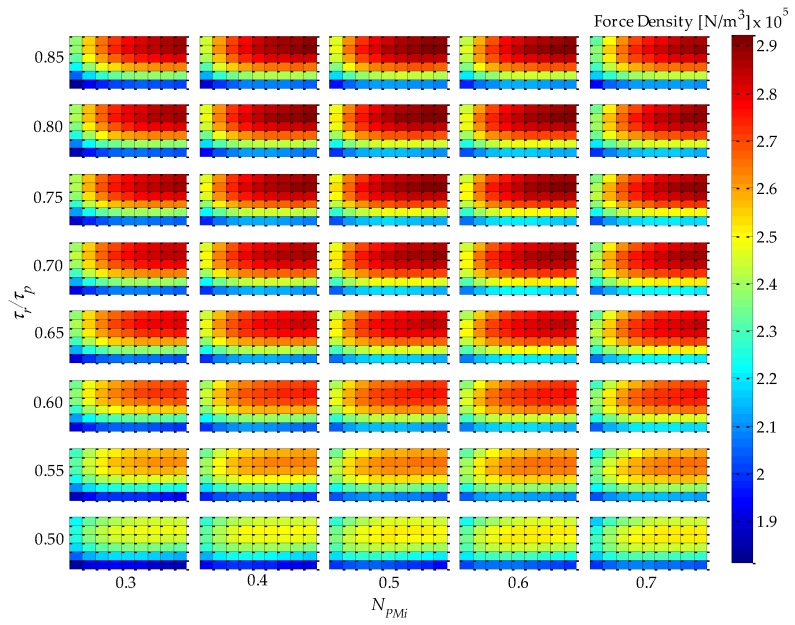
Results of force density for *J_rms_ =* 3 A/mm^2^ and *θ_e_* = π/6, of the uncoupled design for four parametric variables: *τ_r_*/*τ_p_*, *N_PMi_*, *N_CPMs_*, and *n_form_*.

**Figure 5:**
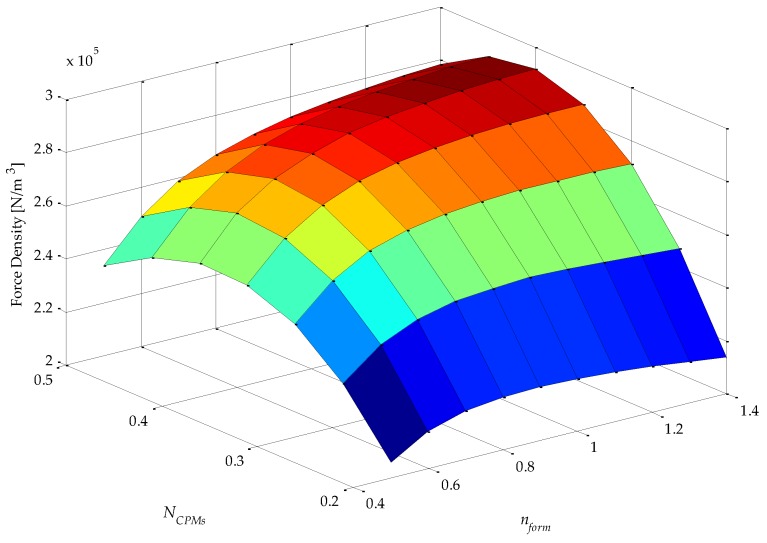
3D graph with results of force density of the uncoupled design for two parametric variables: *N_CPMs_* and *n_form_*. Results for *J_rms_ =* 3 A/mm^2^, *θ_e_* = π/6*,* *τ_r_*/*τ_p_* = 0.75, and *N_PMi_* = 0.60.

Analysis of simulation results using 2D or 3D graphics of a space defined by the four parametric variables presented in Table 1, plus force density and force ripple, can be a difficult and extensive task. An approach that was adopted in this work to analyze results in five dimensions is illustrated in Figure 4. This figure condenses five variables (four parametric variables, which are independent variables, and one objective function variable, which is a dependent variable) on a single graph. It represents a top-oriented view of a combination of 40 3D graphs, in a way that the color map indicates the dependent variable, which, in the case of Figure 4, is force density. The vertical axis holds two independent variables: *τ_r_*/*τ_p_*, which is indicated, and *N_CPMs_* on the minor vertical grid of each rectangle, which is not indicated in Figure 4 so that this figure is not visually overloaded. The horizontal axis also holds two independent variables, *i.e.*, *N_PMi_*, as indicated, and *n_form_* on the minor horizontal grid of each rectangle. Therefore, each of the rectangles in Figure 4 represents a variation of the parametric variables *n_form_*, on the horizontal axis, and *N_CPMs_* on the vertical axis. As an example, a 3D view of one of the rectangles of Figure 4, the one with *τ_r_*/*τ_p_* = 0.75 and *N_PMi_* = 0.60, is shown in Figure 5. The form of presentation of five variables in a single figure allows one to compare results for the entire domain, which represent the combination of all parametric variables according to Table 1, in a comprehensive way.

The force density *F_d_* of Figure 4 and Figure 5 is given by

(10) $$F_{d}=\frac{F_{z1P}}{\pi \left({R_{oPMo}}^{2}-{R_{iPMi}}^{2}\right)\tau_{p}}$$

where *F_z1P_* is the axial force produced by one pole pitch. In the actuator with Halbach arrays, back-irons do not have a significant effect, in fact less than 10% if they are not used at all [11]. Yet, in this work it was decided to keep them to increase performance and for mechanical support. However, they were not taken into account for computation of force density, because *n_BI_* was not considered in the parametric analysis; therefore, there would be no guarantee that force density is maximized if it were computed considering the back-irons.

It should be noted that the shape, or color map, as presented, of force density would not be affected by its initial electrical loading of the uncoupled model, because effective current density is constant; however, its absolute value would be linearly proportional.

From Figure 4, it can be seen that the variable *τ_r_*/*τ_p_* has a greater influence on force density in relation to *N_PMi_*. This can be explained by the increased volume of PMs with radial magnetization as *τ_r_*/*τ_p_* increases, since it leads to higher levels of the radial component of induction in the magnetic gap, while *N_PMi_* slightly alters the total volume of PMs and the distribution of this volume between internal and external PMs. Consequently, the mean radius of the coil is shifted and so its volume is altered; however, this effect is more significantly observed with *N_CPMs_*.

From Figure 4, and with more detail in Figure 5, it is possible to infer that the parametric variable *N_CPMs_* has a higher influence over force density than *n_form_*. This can be explained by a compromise between electrical and magnetic loading, which is directly related to *N_CPMs_*. On the other hand, *n_form_* is related to the interpolar leakage flux, which is more significant for lower values of this parametric variable. A maximum force density of 2.92 × 10^5^ N/m^3^ is obtained with *N_CPMs_* = 0.4 and *n_form_* ≥ 1.2, with a difference of approximately −30% relative to the minimum simulated results observed.

The minimization of force ripple is a desirable feature with regard to the system linearity, reducing problems related to the positioning control and minimizing the inclusion of oscillations by the actuator when the speed is not zero. Depending on the electric drive technique, there are time-dependent induced harmonics that may generate force ripple during dynamic operation; however, these harmonics are not taken into account in this study, once force ripple is computed based on a magnetostatic model.

The force ripple can be estimated statically calculating the difference between the forces obtained with current in 2-phase and 3-phase, corresponding to, e.g., *θ_e_* = 0 and *θ_e_* = π/6, respectively, in relation to the average value between these two cases, according to the following equation:

(11) $$F_{ripple}=2\frac{F_{3}-F_{2}}{F_{3}+F_{2}}$$

**Figure 6:**
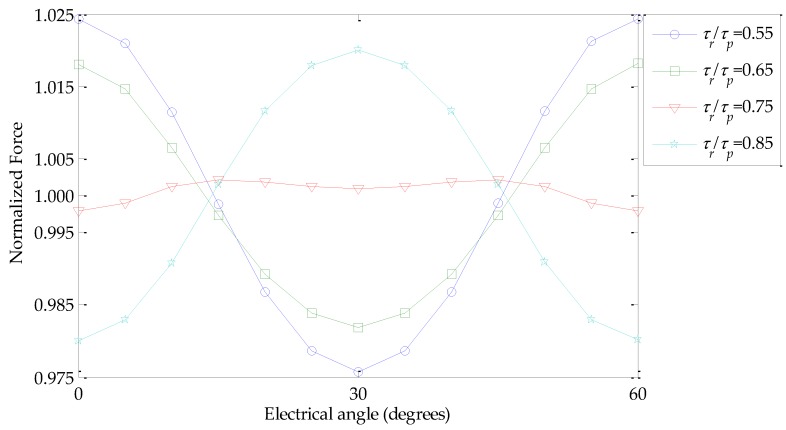
Normalized force computed over a *θ_e_* range of 60 degrees for four *τ_r_*/*τ_p_* variations with *N_CPMs_* = 0.4, *N_PMi_* = 0.6, *n_form_ =* 1.2, and *J_rms_ =* 3 A/mm^2^.

Figure 6 shows an evaluation of force *F*(*θ_e_*) normalized in relation to its mean value computed using its maximum and minimum, *i.e.*, 2*F*(*θ_e_*)/(*F_3_* + *F_2_*). Force results were obtained over a *θ_e_* range of 60 degrees for four *τ_r_*/*τ_p_* variations with *N_CPMs_* = 0.4, *N_PMi_* = 0.6, *n_form_ =* 1.2 and *J_rms_* *=* 3 A/mm^2^. From Figure 6, it is possible to infer that maximum and minimum values of force occur at two particular electrical angles as discussed in the previous paragraph; thus, a fair estimation of the variation of force can be carried out by evaluating *F_2_* and *F_3_* and computing the force ripple according to Equation (11). Figure 6 also shows that for *τ_r_*/*τ_p_* *=* 0.75 the force ripple observed significantly decreased; in fact it is 0.44%, whereas for *τ_r_*/*τ_p_* *=* 0.55 it is 4.87%, *i.e.*, more than 10% higher for the former case.

**Figure 7:**
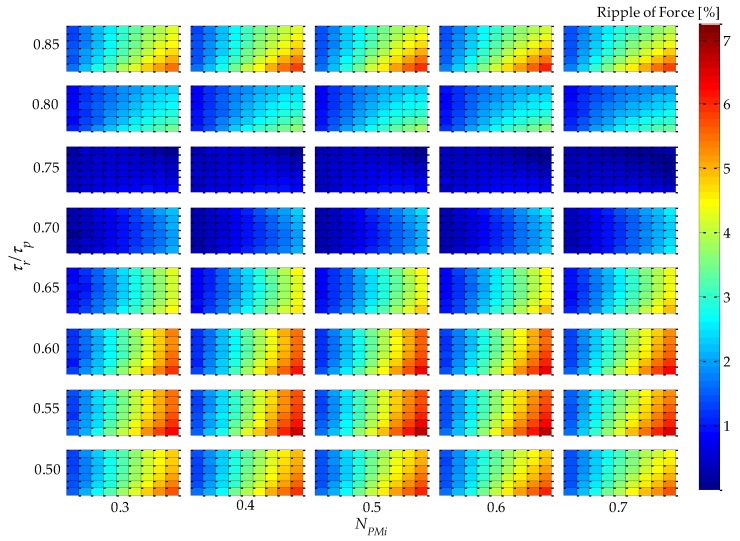
Results of absolute percentage static force ripple of the uncoupled design for four parametric variables: *τ_r_*/*τ_p_*, *N_PMi_*, *N_CPMs_*, and *n_form_*.

Parametric simulation results of *F_3_* and *F_2_* applied to Equation (11) are shown in Figure 7, where the results of absolute value of static force ripple are presented. The results suggest that *τ_r_*/*τ_p_* is closely related to force ripple, and with *τ_r_*/*τ_p_* *=* 0.75 produces approximately zero force ripple regardless of the values of the other variables.

### 3.5. Definition of Dimensional Values for One Pole Pitch of the Uncoupled Design

Based on the result obtained in Section 3.4, and considering the geometrical constraints of Table 2, the dimensional values for geometrical parameters of the actuator in the radial direction can be determined. For this topology, the parametric variables for the uncoupled model chosen were *τ_r_*/*τ_p_* = 0.75, *N_PMi_* = 0.6, *N_CPMs_* = 0.4 and *n_form_ =* 1.2. Results for the uncoupled model are summarized in Section 4. Dimensional results in axial direction are computed in the next subsection.

### 3.6. Computation of Axial Active Length, Total Length, and Number of Poles of the Uncoupled Design

The active volume of the actuator that is able to cope with effective force specifications can be obtained by dividing the rated force *F_r_* (Section 3.1) by the force density *F_d_* obtained for one pole pitch (Section 3.4). The axial active length of the actuator *L_z_* is then calculated by relating it with its active volume, so that

(12) $$L_{z}=\frac{F_{r}}{F_{d}\pi ({R_{oPMo}}^{2}-{R_{iPMi}}^{2})}$$

The axial length of armature *L_zW_* is simply the sum of *L_z_* and the specified stroke *S* (Section 3.1), whereas the total axial length *L_zT_* must be at least the sum of *L_z_* with twice *S*:

(13) $$\left\{ \begin{array}{l} L_{zW}=L_{z}+S\\ L_{zT}=L_{z}+2S \end{array}\right.$$

The number of poles *P* of the actuator can also be determined based on previous results. It was defined that *P* must be an integer even number, and that each of the inner and outer magnets of the extremities count as one pole, even though they present radial length of *τ_r_*/*2*. Therefore, *P* is given by

(14) $$P=2\text{ceil}\left(\frac{L_{z}}{2n_{form}\sqrt{{R_{oPMo}}^{2}-{R_{iPMi}}^{2}}}+\frac{1}{2}\right)$$

where the operator “ceil” rounds the element to the nearest even integer towards positive infinity [19]. In order to obey the condition imposed by Equation (14), *L_z_* or *n_form_* must be recalculated. In this case, it can be observed from Figure 5 that *n_form_* slightly affects *F_d_* over a range of 0.8 up to 1.4. That means it could be adjusted without having a significant effect on the overall results. *n_form_* can be recalculated according to

(15) $$n_{form}=\frac{L_{z}}{(P-1)\sqrt{{R_{oPMo}}^{2}-{R_{iPMi}}^{2}}}$$

It is important to observe that as *n_form_* was recalculated, so must *τ_p_* be, according to Equation (3). If the choice was to adjust *L_z_*, this could lead to an unnecessary increase in the active volume of the device. If *n_form_*, calculated by Equation (15), returns a value outside of the range 0.8–1.4, e.g., it results from the fact that *P* was adjusted to the nearest even number towards positive infinity, but it was very close to an even number towards zero. In this situation, *P* should be set as the closest even number to zero, if the dimensional design conditions verified in the next subsection are met, and the final *n_form_* calculated with Equation (15).

For the case study, values for *L_z_*, *L_zW_*, and *L_zT_* were 116.8 mm, 196.8 mm, and 276.8 mm, respectively. *P* was found to be 4 with a final *n_form_* of 1.1633.

### 3.7. Validity of Parameters of the Uncoupled Design

This step in the designing process verifies whether the results obtained so far are consistent in the sense that the final shape of the actuator presents acceptable dimensions and parameters, which are defined by the following inequalities:

(16) $$\left\{ \begin{array}{l} \frac{L_{z}}{L_{zW}}\geq 0.5\\ P\geq 4\\ L_{rPMi}\geq 3\text{ mm}\\ L_{rPMo}\geq 3\text{ mm} \end{array}\right.$$

The first inequality, which is the ratio between the axial active length and the windings axial length, is set so that at least half of the total length of the windings is active during operation. This ratio is related to the efficiency of the actuator, once there are Joule losses along the total length of the windings, but only the portion that is placed within the active length produces force.

On the other hand, the second inequality in Equation (16) requires that the minimum number of poles of the actuator is four. This criterion is established in order to limit the way that end effect affects the overall performance of the actuator. End effect is intrinsic to linear machines and is more significant if the device has a low number of poles.

**Figure 8:**
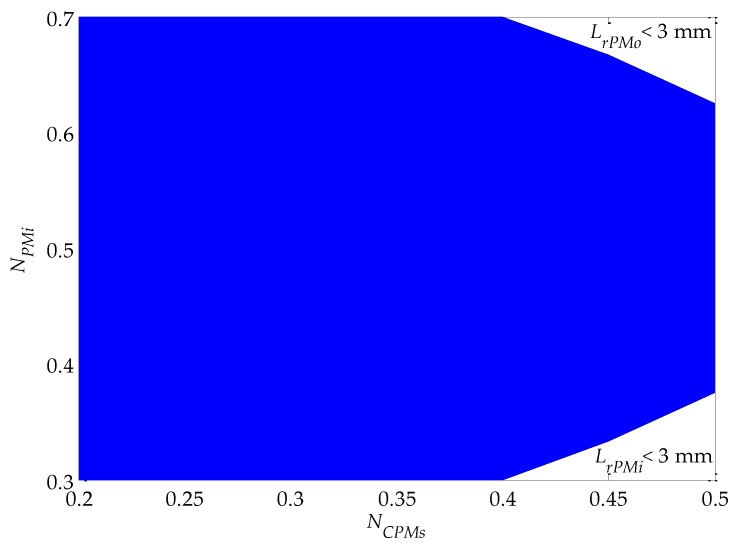
Limitation of the radial length of the PMs given by inequalities 3 and 4 in Equation (16) as a function of the parametric variables *N_PMi_* and *N_CPMs_*, where *L_rPMi_* = (*R_oPMi_* − *R_iPMi_*) is the radial length of the inner PMs and *L_rPMo_* = (*R_oPMo_*–*R_iPMo_*) is the radial length of the outer PMs.

The third and fourth inequalities of Equation (16) limit the radial length of the permanent magnets to a minimum, which are imposed to prevent the PMs from breaking during assembly, which would easily happen if the radial length of the PMs is too small. The shaded area in Figure 8 indicates a domain of the parametric variables *N_PMi_* and *N_CPMs_* in which radial length of both inner and outer PMs are bigger than 3 mm, whereas the areas that are not shaded indicate whether inner or outer magnets present a radial length smaller than specified.

For the case under study, it was found that *P* = 4 and *L_z_*/*L_zW_* = 0.59; thus, both criteria were simultaneously met in the first place and no change in the geometrical constraints was required. If, for example, the resultant *L_z_*/*L_zW_* were lower than 0.5, then geometrical constraints ought to be reviewed, as the first inequality of Equation (16) would not be complied with. In this case whether *R_oPMo_* should be decreased or *R_iPMi_* increased, so that the active axial length of the actuator would become larger. The radial length of the inner and outer PMs is also higher than 3 mm, once in Section 3.4 *N_PMi_* and *N_CPMs_* were defined as 0.6 and 0.4, respectively, which, according to Figure 8, lies within the allowed domain. If the former discussed conditions were not attained, it would be suggested to alter *N_PMi_*, which presents very little sensitivity in relation to design objective, instead of changing *N_CPMs_*.

The inequalities given by Equation (16) could be different from the ones that were set; they are basically criteria imposed by the designer.

### 3.8. Definition of Thermal Topology

The heat sources associated with losses of an actuator can be of many kinds; however, in this particular actuator, the main source of losses is Joule losses at the conductors. Very low levels of induced current appear in the PMs, which can be neglected. This specific topology presents an intrinsic low level of iron losses because there is no relative movement between back-irons and PMs and it presents a coreless armature. Still, there may be induced current in the back-irons by variation of the flux produced by the armature, but in lower levels than would be observed within actuators with cored armature and low compared to Joule losses, so it can be neglected. Friction losses at bearings are also not considered.

In order to achieve higher force and power density, the device must present an improved capacity of heat exchange. In the proposed methodology, thermal analysis plays an important role, since it allows for determining absolute force density and, thereafter, active volume for a given specification.

In this work, it was considered that a forced air flow was imposed to a hollow shaft, which insufflates the actuator with air at ambient temperature. In order to improve heat exchange in this topology, a forced inlet flow with a speed of 0.5 m/s enters the hollow shaft from the bottom, passes through the inner gap, then passes through the outer gap and outflows at the top of the actuator with a higher temperature. This characterizes a forced convection at the hollow shaft and at the inner and outer gaps, which significantly improves heat transfer.

It must be clear that the decision about whether forced or natural heat transfer is applied must be made in accordance with the mechanical characteristics of the actuator. Depending on the operation conditions, the actuator may be completely sealed, or, in a different situation, it might be open and its own movement produces an air flow that characterizes forced convection. In any case, it must be defined at this step because this significantly affects the designing process.

### 3.9. Building and Simulation of Thermal 2D FEM of the Full Axial Length of the Actuator

The thermal analysis was performed using FEA by means of the commercial package ANSYS Fluent^®^. The forms of heat transfer considered in the simulation were by radiation, by conduction, and by forced and natural convection. The models for convection and radiation used were Boussinesq and Rosseland, respectively, which are appropriate for this topology [20]. For the given air flow, the Reynolds number is relatively low, in the order of 500 [21]; therefore, a laminar flow can be considered.

Additionally, some important assumptions were made: an atmosphere temperature of 300 K is set; results are for steady state condition; gravity is 9.81 m/s^2^; the model is axisymmetric; the actuator operates with its moving axis at a vertical position; and the temperature of the windings was set as the maximum allowable temperature (discussed in Section 3.9.2), *i.e.*, 353 K.

**Figure 9:**
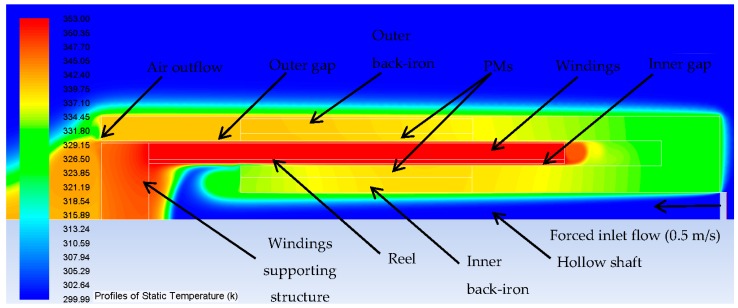
Results of 2D axisymmetric thermal simulation of the full length of actuator design with dimensions found for the uncoupled model but with a constant temperature of 353 K at the windings.

The profile of static temperature over the actuator, considering thermal properties of materials and temperature of the windings (presented in Section 3.9.1 and Section 3.9.2, respectively), resultant from the simulation is shown in Figure 9. Although the axial length of the actuator is presented in a horizontal position here, for convenience, gravity was set parallel to the axial direction.

#### 3.9.1. Thermal Material Properties

The thermal properties of the materials of which the actuator is composed and properties of the air that were considered to perform the simulation presented in Section 3.10 are given in Table 3. These properties were obtained from the Ansys Fluent^®^ library.

#### 3.9.2. Windings’ Maximum Allowable Temperature

The maximum allowable temperature of the conductors at full load could be determined according to their insulation class. Nevertheless, if the conductor’s maximum temperature is respected, the maximum operation temperature of the PMs should also be respected. Sintered NdFeB PMs with a nominal maximum energy product of 278 kJ/m^3^ and a maximum operation temperature of 80 °C represent one of the worst cases of such materials commercially available [22]. This material specification results in an actuator with the lowest force density, thus the device’s inferior performance limit can be assessed. For this reason, the maximum allowable temperature at the windings was set as 80 °C. As there is no physical contact between windings and PMs, and considering the forced convection present in the inner and outer air-gap, setting the windings temperature as 80 °C guarantees that PMs temperature will not reach its maximum. The temperature must be set at the windings because that is the heat source and this allows for a proper simulation of the heat exchange phenomena.

### 3.10. Permanent Magnets Mean Temperature

The mean operating temperature of the PMs at full load can be obtained from the thermal simulation presented in Section 3.10. From the simulation, it can be observed that there is a low variance of the distributed temperature over the PMs, which results from the fact that they present relatively high thermal conductivity. Results showed that the mean temperature of the inner PMs is 66 °C, whereas the mean temperature of the outer PMs is 68 °C.

In the case of actuators that do not employ PMs, this step of the design process and the one in Section 3.11 should be neglected, e.g., for reluctance linear motors.

### 3.11. Calculation of Thermal Corrected H_c_ and B_r_

Sintered NdFeB permanent magnets present a high *B*-*H* energy product, a recoil permeability that can be considered constant, a magnetic permeability similar to free space, a high coercive field, and a high remanent induction. These characteristics are desired in order to produce an actuator with elevated force density; however, the properties of the PMs are significantly affected by temperature and this should be considered in the design process. Manufacturers specify that both *H_c_* and *B_r_* can be corrected as a function of the operation temperature in a linear way while operating under the maximum allowable temperature [22]. For the PMs employed in this case study, *B_r_* is reduced by 0.12%/°C, $\alpha_{Br}$ = 0.0012, and *H_c_* is decreased by 0.7%/°C, $\alpha_{Hc}$ = 0.007, according to

(17) $$\left\{ \begin{array}{l} B_{r}(T)=B_{r}(T_{0})[1-\alpha_{Br}(T-T_{0})]\\ H_{c}(T)=H_{c}(T_{0})[1-\alpha_{Hc}(T-T_{0})] \end{array}\right.$$

Considering the operating temperature of inner and outer PMs obtained at the preceding subsection, the properties *H_c_* and *B_r_*, and, consequently, the relative permeability *µ_r_*, are given in Table 4.

It should be observed that as the operating temperatures of inner and outer magnets are slightly different from each other, the properties of the PMs are also slightly differently affected. In a topology such as the dual-Halbach array actuator, this could lead to an optimal design with a higher volume of inner or outer PMs, *i.e.*, performance could be affected by the parametric variable *N_PMi_*, especially if another thermal exchange method was applied, and it would lead to more significant differences between the operating temperature of the PMs.

The PM properties presented in Table 4 must be corrected at the 2D FEM for one pole pitch, so that together with the maximum effective current density, an absolute value of force density can be calculated in which thermal and geometrical constraints are taken into account.

### 3.12. Determination of the Overall Heat Transfer Coefficient

It is necessary to determine the overall heat transfer coefficient because it directly affects the maximum effective current density that can be applied to the windings in order to keep the maximum temperature within limits.

**Figure 10:**
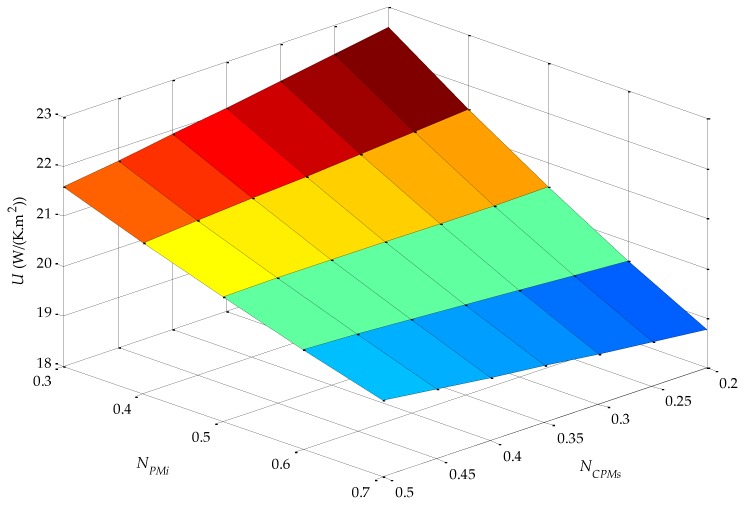
Overall heat transfer coefficient as a function of the parametric variables *N_PMi_* and *N_CPMs_*.

With systems that present composite forms of heat transfer, such as the actuator under study, it is often convenient to work with an overall heat transfer coefficient *U*, which is defined by an expression analogous to Newton’s law of cooling [21]:

(18) $$q_{x}=UA_{s}\Delta T$$

where Δ*T* is the overall temperature difference, *A_s_* is the heat exchange surface area and *q_x_* is the heat rate (W). Results obtained from Section 3.10 allow for calculating *U* and are presented in Figure 10.

The parametric variable *τ_r_*/*τ_p_* does not affect *U* because it does not alter the volume of electric conducting material. On the other hand, *n_form_* affects the volume of conducting material for one pole pitch because axial length of one pole varies with *n_form_*. Even so, this variable does not affect the radial length of conductors and PMs and, moreover, once the total active volume of the actuator is determined (Section 3.18), *n_form_* is used to define the number of poles of the device. Thus, it should be noted that *U* only varies with the parameters *N_PMi_* and *N_CPMs_*, as shown in Figure 10.

### 3.13. Maximum Effective Current Density

The Joule losses *P_Joule_* produced by the windings can be determined by

(19) $$P_{Joule}=V_{w}F_{f}\rho {J_{rmsMax}}^{2}$$

where *V_w_* is the total volume of the windings, *F_f_* is the windings fill factor, *ρ* is the resistivity of the copper, and *J_rmsMax_* is the maximum effective current density at the windings maximum allowable temperature. As only Joule losses are considered, since other kinds of losses are negligible, the heat rate is equal to *P_Joule_*; thus, the maximum effective current density is given by

(20) $$J_{rmsMax}=\sqrt{\frac{UA_{s}\Delta T}{V_{w}F_{f}\rho }}$$

**Figure 11:**
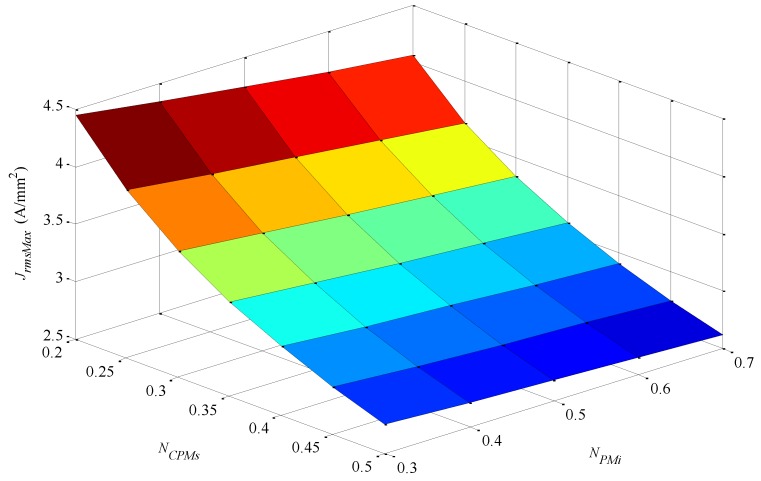
Maximum effective current density applicable at the windings in order to reach the maximum allowable temperature of 80 °C at the windings as a function of the parametric variables *N_CPMs_* and *N_PMi_*.

Only the parameters *N_PMi_* and *N_CPMs_* affect *J_rmsMax_*, for an equivalent reason to that explained for *U*. A 3D plot of *J_rmsMax_* is shown in Figure 11.

Maximum effective current density is observed for parameters *N_PMi_* = 0.3 and *N_CPMs_* = 0.2, which correspond to the relatively lowest volume of windings. On the other hand, with *N_PMi_* = 0.7 and *N_CPMs_* = 0.5, the design presents the relatively highest volume of windings. In short, designs with a relatively low volume of windings allow higher levels of current density, while the opposite is true for designs with relatively high volume of windings. This is explained by the fact that a higher volume of windings would produce more Joule losses, whereas its overall heat transfer coefficient does not increase at a proportional rate, so its effective current density must be proportionally adjusted.

### 3.14. Simulation of Quasi-Static Parametric 2D FEM for One Pole Pitch

The quasi-static parametric model discussed in Section 3.3 can be used at this step. The only modification should be the parameters *B_r_* and *H_c_* of the inner and outer magnets, based on the results presented in Table 4. This approach provides a fast and quite accurate approximation once very little variation of the temperature of the PMs is observed due to the fact that there is no change in the radial length of the air-gaps and in the maximum allowable temperature of the windings while performing parametric simulation. There is a slight difference in the air flow through the inner and outer gap associated with parameters *N_CPMs_* and *N_PMi_* once those modify the volume of the air-gap. It was observed from the simulation that this difference in the air-gap volume alters the mean temperature of the magnets by less than 3%.

From the flowchart of Figure 2, it is possible to observe that the maximum effective current density for each design is not an input of this step. This results from the fact that force density has a linear relationship with current density. So, in order to simplify the analysis, the model was simulated with 1 A/mm^2^, and, thereafter, the absolute maximum force density for each design was obtained in a post-process, simply by multiplying each corresponding value following the results obtained in Section 3.13. This is done as a post-process, in order to speed up the method.

If results do not meet the specific design criteria, assessed in Section 3.18, the geometrical constraints for one pole pitch must be reviewed. For this case study, parameters *R_oPMo_* or *R_iPMi_* should be altered and this should be applied to the thermal and electromagnetic models, as indicated in Figure 2.

### 3.15. Electromagnetic Coupled Analysis

A similar approach to the one presented in Section 3.4 is carried out at this step, *i.e.*, first, the results for force density are evaluated as a function of the parametric variables *τ_r_*/*τ_p_*, *N_PMi_*, *N_CPMs_*, and *n_form_*. After considering the ripple of force, the ratio *τ_r_*/*τ_p_* is chosen and, considering the geometrical constraints, *N_PMi_* is defined. A 3D graph of force density for the chosen *τ_r_*/*τ_p_* and *N_PMi_* is presented detailing results as a function of the parametric variables *N_CPMs_* and *n_form_*.

**Figure 12:**
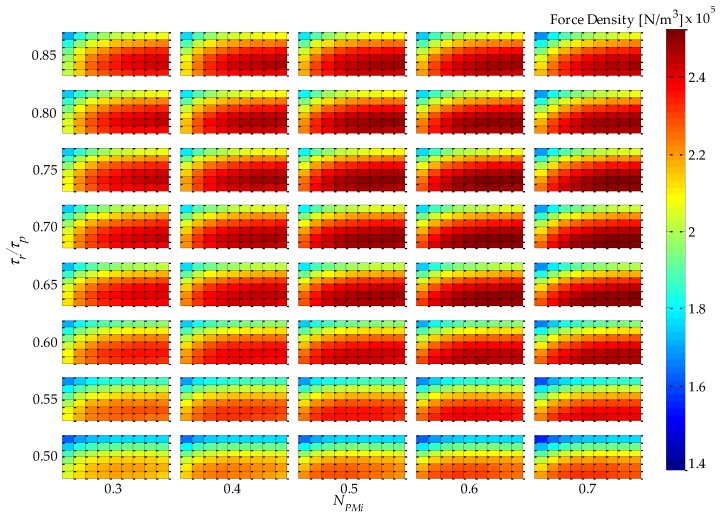
Results of force density for effective current density according to Figure 11 of the coupled design for four parametric variables: *τ_r_*/*τ_p_*, *N_PMi_*, *N_CPMs_*, and *n_form_*.

It is important to reinforce that, at this stage, effective force density is corrected in a post-process routine using Matlab^®^ script for the reasons discussed in the previous subsection. Each design evaluated presents a maximum effective current density, resulting in the maximum allowable temperature at the windings. This means that the force density values presented in Figure 12 are the absolute maximum considering energy drop at the PMs and thermal constraint.

**Figure 13:**
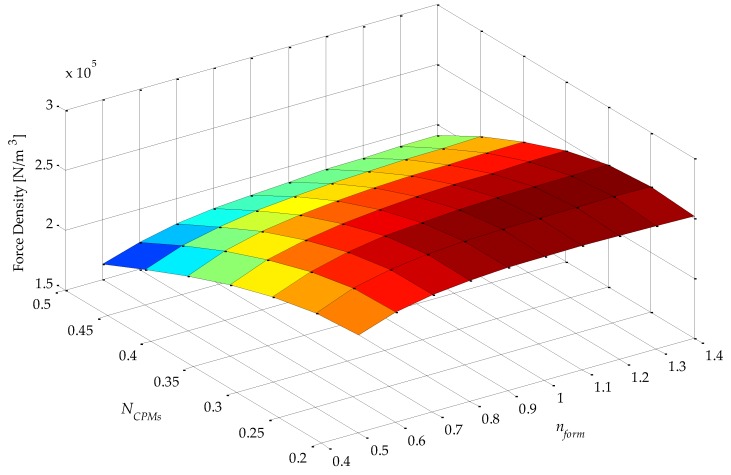
3D graph with results of force density of the coupled design for two parametric variables: *N_CPMs_* and *n_form_.* Results for *τ_r_*/*τ_p_* = 0.75 and *N_PMi_* = 0.60.

The results presented in Figure 12 are significantly different from those presented in Figure 4, especially because of the parametric variable *N_CPMs_*, which is directly related to the volume of the windings. It is important to emphasize that in Figure 4 the force density for the uncoupled model is obtained with constant effective current density; on the other hand, in Figure 12, in the coupled model, the effective current density is adjusted according to Equation (20). This becomes even more evident when comparing Figure 5 and Figure 13, where the independent parametric variables are clearly identified, and the force density dependency shows that there is a shift in the maximum point in relation to *N_CPMs_*, from 0.4 for the uncoupled model to 0.25 for the coupled model. The parametric variable *N_CPMs_* directly affects the volume *V_w_* and external surface area *A_s_* of the windings, and these are used to calculate *J_rmsMax_*.

Moreover, the absolute maximum force density has also changed from 2.92 × 10^5^ N/m^3^ for the uncoupled model to 2.56 × 10^5^ N/m^3^ for the coupled model. This will directly affect the total active volume of the actuator and, consequently, its dimensional parameters. Even though the figures of maximum force density are close to each other, in this case, they result from a good estimation of the maximum electrical loading. The results could significantly differ, e.g., if: the initial effective current density were set as 5 A/mm^2^ for the uncoupled model, which is often employed in conventional electrical machines; if the maximum allowable temperature at the windings were set as 120 °C, which in general is acceptable because of its insulation class; or if the NdFeB PMs operation temperature were higher.

**Figure 14:**
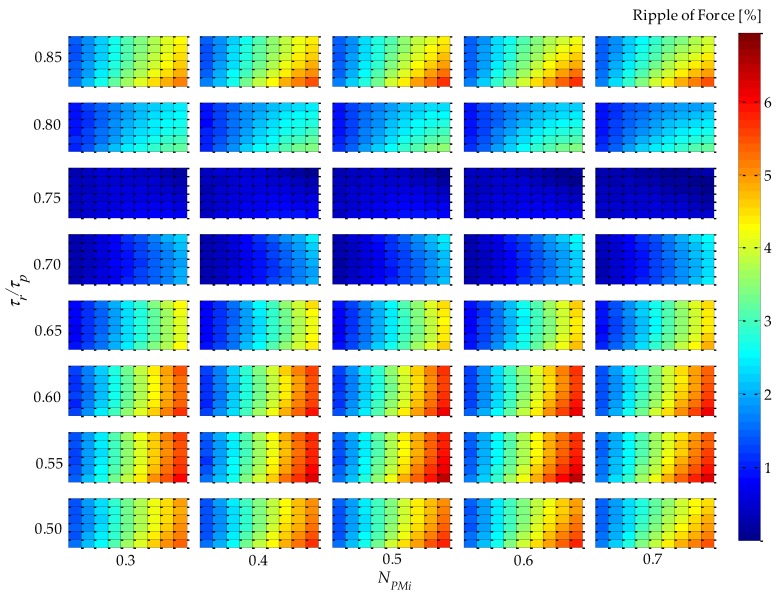
Results of absolute percentage static force ripple of the coupled design for four parametric variables: *τ_r_*/*τ_p_*, *N_PMi_*, *N_CPMs_*, and *n_form_*.

The absolute force ripple presented in Figure 14 shows behavior similar to that in Figure 7, *i.e.*, for *τ_r_*/*τ_p_* = 0.75, the percentage ripple of force is approximately zero. These results were expected because this parametric variable is not affected by thermal issues because it does not alter the volume of the windings or the volume of the PMs.

### 3.16. Definition of Dimensional Values for One Pole Pitch of the Coupled Design

Based on the results obtained in Section 3.15, and considering the geometrical constraints given in Table 2, the dimensional values for the geometrical parameters of the actuator in the radial direction can be determined. The parametric variables chosen were *τ_r_*/*τ_p_* = 0.75, *N_PMi_* = 0.6, *N_CPMs_* = 0.25, and *n_form_* *=* 1.0. Applying the chosen parametric variables to the equations in Section 3.3 in conjunction with the parameters obtained in the next section, it is possible to finalize the design for the coupled model in a similar way as for the uncoupled model. Results for the coupled model are summarized and compared to the coupled model in Section 4.

### 3.17. Computation of Axial Active Length, Total Length, and Number of Poles of the Coupled Design

This step is the same as in Section 3.6, except for the fact that this time the results of the parametric variables are based on the coupled model.

For the case study, values for *L_z_*, *L_zW_*, and *L_zT_* were 133.2 mm, 213.2 mm, and 293.2 mm, respectively. *P* was found to be 6 with a final *n_form_* of 0.7961.

### 3.18. Verification if Parameters of the Coupled Design Are Valid

The same criteria as adopted in Section 3.7 were applied at this step. For the coupled model, it was found that *p* = 6 and *L_z_*/*L_zW_* = 0.624; thus, those two criteria were simultaneously met. The radial length of the inner and outer PMs is higher than 3 mm, once in Section 3.15 *N_PMi_* and *N_CPMs_* were defined as 0.6 and 0.25, respectively. That, according to Figure 8, lies within the allowed domain.

If any of the criteria given by Equation (16) are not met, the geometrical constraints should be reviewed; this ought to be applied to the thermal and electromagnetic coupled model in order to perform a second coupled analysis.

## 4. Results and Discussion

This section discusses the influence of each parametric variable studied for the case under study; it also presents the final designs for the coupled and uncoupled models and compares the results.

For the topology under study, it was found that the parametric variable *τ_r_*/*τ_p_* presents high sensitivity in relation to the ripple of force. Setting *τ_r_*/*τ_p_* = 0.75 was sufficient to take the ripple of force practically to zero, regardless of the value of the other parametric variables. It should be observed that the value of *τ_r_*/*τ_p_* for which the ripple of force becomes close to zero may change for different geometrical constraints. Differences between uncoupled and coupled models were found to be insignificant regarding how *τ_r_*/*τ_p_* affects the ripple of force.

The parametric variable *N_PMi_* slightly affected the design objectives, *i.e.*, high force density and low ripple of force. Results for *N_PMi_* from 0.5 up to 0.7 were almost the same. In this case, an *N_PMi_* value that leads to a relatively lower volume of magnets could be applied, which, in summary, represents setting a higher value for *N_PMi_*. However, *N_PMi_* is limited by the constraint related to the minimum radial length of the PMs. Also, as discussed in Section 3.11, *N_PMi_* could become more relevant for the coupled model if the differences between the temperature of inner and outer PMs were higher; thus, the PMs with lower temperature should present relatively greater volume, which can be achieved by adjusting *N_PMi_*.

The most relevant parametric variable in relation to force density is *N_CPMs_*, especially when the coupled and uncoupled models are compared to each other. For the uncoupled model, with a constant current density no matter the volume of the active windings, force density was maximized for *N_CPMs_* equals to 0.4. On the other hand, with the coupled model, in which the effective current density is corrected in order to keep the temperature in the actuator limited to its specified maximum, force density was best when *N_CPMs_* is equal to 0.25. In a simple way, this implies that, if temperature limits are considered, the electrical loading is decreased in detriment of an increase in magnetic loading, once Joule losses are limited.

The parametric variable *n_form_* plays an important role in defining the number of poles *P* of the actuator. In both the uncoupled and coupled models, it is also relevant to find the best force density; nevertheless, it showed low sensitivity in the range 0.8 to 1.4. For low values of *n_form_*, such as 0.4, there is significant leakage flux between adjacent poles because these poles become close to each other, thus reducing force density. The low sensitivity observed helps in the designing process because *n_form_* can be defined in a wide range so that the number of poles can be set with a pre-defined axial active length.

**Figure 15:**
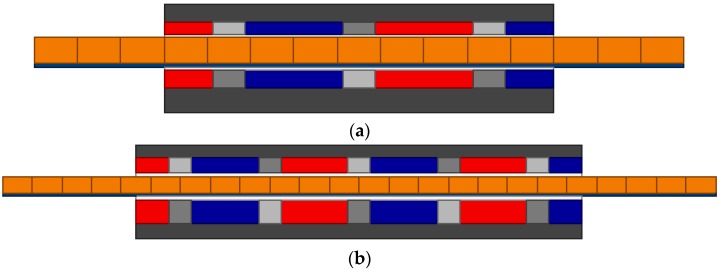
Axisymmetric view of the electromagnetic actuator for: (**a**) the uncoupled model with *P* = 4, *N_CPMs_* = 0.4, *N_PMi_* = 0.6, *τ_r_*/*τ_p_* = 0.75, and *n_form_* = 1.1633, and *n_BI_* = 0.4; (**b**) coupled model with *P* = 6, *N_CPMs_* = 0.25, *N_PMi_* = 0.6, *τ_r_*/*τ_p_* = 0.75, = *n_form_* = 0.7961, and *n_BI_* = 0.4.

According to the proposed methodology, the number of poles and final dimensions of the actuator are those found for the coupled model, although the results of the uncoupled model are indispensable to give a first approximation in order to enable thermal analysis. For the sake of comparison, Table 5 presents the number of poles and the dimensions of each design, while Figure 15 presents the two axisymmetric finite element models, showing their final shape.

Temperatures in the actuator of the coupled model are those presented in Figure 9, once the effective current density was adjusted to obtain those results. The maximum effective current density for the coupled design is 3.71 A/mm^2^, which is higher than initially estimated; however, it should be noted that there is a lower volume of windings than previously mentioned. Finite element simulation of the axisymmetric model of Figure 15b resulted in an axial force of 121.8 N, which is close to the specifications, *i.e.*, 120 N. These results show that the end effect has little significance, which is consistent with the assumption that PMs with radial magnetization at the end poles should present an axial length of *τ_r_*/2, as expected.

In contrast, temperatures for the uncoupled design were simulated with a current density of 3 A/mm^2^. In this case the mean temperature of the windings was approximately 90 °C, whereas the mean temperature was approximately 74 °C for the inner PMs and approximately 76 °C for the outer PMs. The temperatures at the PMs are close to the maximum specified by the manufacturer, *i.e.*, 80 °C [22], which can easily lead to demagnetization, especially if high transitory currents are applied during operation.

A FEM simulation of the axisymmetric uncoupled model shown in Figure 15a, with 3 A/mm^2^ without considering temperature effect over PMs, *i.e.*, with *B_r_* = 1.23 T and *H_c_* = −890 kA/m, resulted in 120.4 N axial force. Now, if the same current density is applied, but the PMs’ remanent induction and coercive force are adjusted according to their temperature, as given by Table 6, the axial force produced by the actuator is 92.5 N. This result shows the relevance of temperature to the PMs and, thus, to the performance of the actuator, once axial force decreased by 22.9% in relation to specification.

In fact, the uncoupled model could operate at the same temperature as the coupled one; however, in this case, the maximum effective current density should be 2.75 A/mm^2^. Considering this effective current density and adjusting PMs parameters according to their operating temperature, presented in Table 4, the axial force produced with this design drops to 89.9 N, which represents a 25% reduction when compared to the specifications.

**Figure 16:**
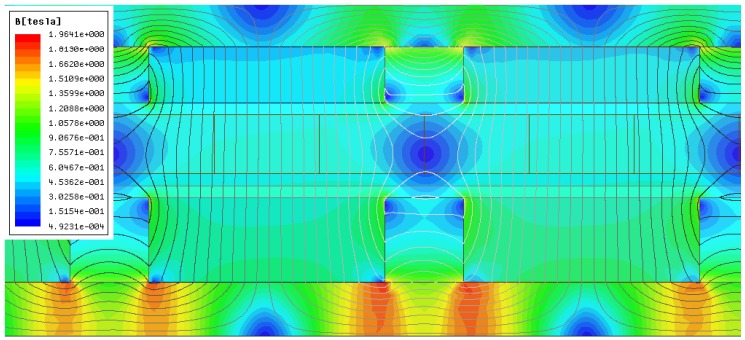
Magnitude of magnetic induction and flux lines of a section of the final actuator design with nominal effective current density.

The magnetic induction of the final actuator design (shown in Figure 15b), evaluated via finite element analysis, is shown in Figure 16. It can be observed that saturation is not reached in the back-irons, although the inner back-iron presents a higher magnitude of induction when compared to the outer one. This suggests that inner and outer back-irons could be adjusted independently from each other. It should be noted that the results shown in Figure 16 were obtained by applying a nominal effective current density; therefore, small levels of asymmetry in the flux lines and in the distribution of magnetic induction are caused by armature reaction. In terms of magnitude, the peak value of the induction in the middle of the magnetic gap is approximately 0.55 T, whereas it is nearly 1.96 T in the inner back-iron.

**Figure 17:**
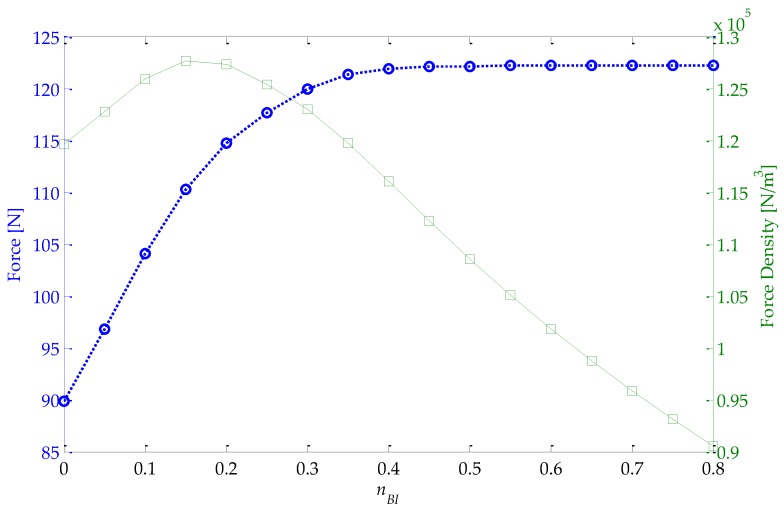
Force and force density of the final actuator design as a function of the parametric variable *n_BI_* obtained with nominal effective current density.

It is mentioned in Section 3.3.3 that the volume of back-irons may be optimized after the active volume of the coupled model is determined. The design methodology was carried out considering *n_BI_* = 0.4; however, in Figure 17, the force and force density of the final design of the actuator, according to Figure 15b, were obtained as a function of *n_BI_*. The force density, in this case, is computed by considering a volume given by$\pi \left({R_{o}}^{2}-{R_{i}}^{2}\right)L_{z}$.

It can be observed that for *n_BI_* = 0 the total force is approximately 90 N, while for *n_BI_* = 0.8 the axial force is 122 N. More importantly, with *n_BI_* = 0.4 there is almost no force drop in relation to its maximum. For *n_BI_* smaller than 0.4, a magnetic potential drop is present in the back-irons, which is reflected as a reduction in the radial component of magnetic induction in the magnetic gap, resulting in lower levels of force.

On the other hand, force density was found to be maximized for *n_BI_* = 0.15, with approximately 1.28 × 10^5^ N/m^3^. Nevertheless, at maximum force density, force is equal to 110.2 N, which does not meet the specification of 120 N. Force density decreases significantly for larger values of *n_BI_* once the volume of the actuator increases and force is almost constant.

In order to comply with the force requirement without compromising force density, *n_BI_* could be set as 0.3, nearly 120 N. Thus, it implies that *R_i_* = 14.69 mm and *R_o_* = 40.90 mm.

The presented design methodology was shown to be effective in designing a dual-Halbach array linear actuator with thermal-electromagnetic coupling, once the results obtained for the final design meet the specifications given in Section 3.1 while the geometrical constraints of Section 3.9.1 and the thermal constraints of Section 3.9.2 are considered.

The main strengths of the design methodology presented can be highlighted as follows: it provides a step-by-step method for designing an actuator so that its overall dimensions can be obtained based on a one pole pitch electromagnetic analysis, which is simple and fast to compute; it applies thermal-electromagnetic coupling, which allows one to obtain simulation results that are expected to be in good agreement with experimental ones; and it considers thermal constraints applied to the windings, ensuring that the maximum temperature is not exceeded on either PMs and windings, by means of the specification of a maximum effective current density for continuous operation with safety.

The main drawbacks of the methodology are as follows: the use of parametric analysis is a quite extensive work, even though simulation can be fast for 2D models; the design procedure can be laborious if the parameter verification defined by inequalities given in Section 3.7 and Section 3.18 is not met in the first place; and the final design is not necessarily optimal, because parametric simulation is a discrete domain, although the result can be very close to optimal if discretization of the parametric variables with higher sensitivity is increased.

## 5. Conclusions

The methodology presented is effective and allows one to obtain in a step-by-step manner a final design considering thermal and geometrical constraints. Such a methodology is an important tool to assist in the design process of linear cylindrical actuators, and its general concepts could be applied to a wide range of actuator topologies.

The results of the study showed that if the thermal effect is not considered, the device may not meet the specifications; nonetheless, in order to meet the specifications, operating temperatures may exceed the maximum values specified for the materials.

It is important to note that, when considering electromagnetic-thermal coupling, the optimum design can be significantly different from a model that only considers electromagnetic behavior, especially with regard to the ratio between the volume of windings to the volume of permanent magnets.

Further work should produce analytical models for thermal and electromagnetic analysis and apply a mathematical optimization method in order to find a global maximum in an automatic way instead of performing parametric analysis. The inner and outer back-irons may also be taken into account separately in an optimization process in a future work.

## Figures and Tables

**Table 1:** Design variables evaluated for the case study.

Variable	Evaluated Values	Step
*N_CPMs_*	0.2–0.5	0.05
*N_PMi_*	0.3–0.7	0.1
*τ_r_*/*τ_p_*	0.5–0.85	0.05
*n_form_*	0.4–1.4	0.1

**Table 2:** Design constraints of the case study.

Variable	Evaluated Values
Inner radius of internal PMs (*R_iPMi_*)	18 mm
Outer radius of external PMs (*R_oPMo_*)	38 mm
Inner and outer mechanical gap (*M_Ci_* and *M_Co_*)	1 mm
Radial length of Reel (*L_rReel_*)	1 mm
Radial length of inner and outer PMs	≥3 mm
Back-iron factor (*n_BI_*)	0.4

**Table 3:** Thermal properties of actuator materials and air used in thermal simulation.

Material	Density (kg/m³)	Specific Heat(J/kg-K)	Conductivity (W/m-K)	Emissivity	Viscosity (kg/m-s)	Thermal Expansion Coefficient (1/K)
NdFeB	7500	502	7.7	0.82	-	-
Steel	8030	502.48	16.27	0.65	-	-
Teflon	2200	970	0.35	0.87	-	-
Air	1.125	1006.43	0.0242	-	0.000017894	0.00335
Copper	8978	381	387.6	0.68	-	-

**Table 4:** Corrected values of inner and outer PM properties as a function of operating temperature.

Property	Inner PMs	Outer PMs
*B_r_* [T]	1.169	1.166
*H_c_* [kA/m]	−635	−622
*µ_r_* [-]	1.464	1.491

**Table 5:** Number of poles and dimensional results for the uncoupled and the coupled model. Dimensions are presented in mm.

Design	*P*	*R_i_*	*R_oPMi_*	*R_iReel_*	*R_iC_*	*R_oC_*	*R_iPMo_*	*R_o_*	*τ_r_*	*τ_z_*	*L_z_*	*L_zW_*	*L_zT_*
Uncoupled	4	10.66	23.4	24.4	25.4	33.4	34.4	43.45	29.19	9.73	116.8	196.8	276.8
Coupled	6	13.42	25.2	26.2	27.2	32.2	33.2	41.81	19.98	6.66	133.2	213.2	293.2

**Table 6:** PM properties of the uncoupled model, adjusted according to their operating temperature with electrical loading of 3 A/mm^2^.

Property	Inner PMs	Outer PMs
*B_r_* [T]	1.158	1.155
*H_c_* [kA/m]	−585	−572
*µ_r_* [-]	1.575	1.607

## Abbreviations

The following abbreviations are used in this manuscript:

PMs: Permanent magnets
NdFeB: Neodymium Iron Boron
DC: Direct current
FEM: Finite element model
FEA: Finite element analysis
